# Experimental evaluation on the applicability of necrobiome analysis in forensic veterinary science

**DOI:** 10.1002/mbo3.828

**Published:** 2019-03-12

**Authors:** Fabiola Tuccia, Emad Zurgani, Sara Bortolini, Stefano Vanin

**Affiliations:** ^1^ Department of Biological and Geographical Sciences, School of Applied Sciences University of Huddersfield Huddersfield United Kingdom; ^2^ Department of Life Sciences University of Modena and Reggio Emilia Reggio Emilia Italy; ^3^ Gruppo Italiano per l'Entomologia Forense (GIEF) Italy; ^4^ Present address: Faculty of Science and Technology Free University of Bozen‐Bolzano Bozen‐Bolzano Italy

**Keywords:** forensic microbiology, forensic veterinary, necrobiome, postmortem microbiome

## Abstract

Despite the wide usage of animals as models in forensic studies, the investigations of fundamental legal questions involving domesticated and nondomesticated animals were always given marginal attention compared to “human forensic,” and only recently the interest in the discipline is increasing. Our research focuses on the effect of the fur coat on the activity and development of microbial decomposers. In order to test this variable never assessed before, rabbit carcasses were used and results show that: (i) distinct and significant temporal changes in terms of metabolic activity and taxa distribution can be tracked over the decomposition process; (ii) the richness and the diversity of the bacterial communities does not significantly vary over time, but it does not mean that the species Operational Taxonomic Units (OTUs) do not change; (iii) the presence/absence of the fur on the carcasses does not significantly affect either the bacterial communities’ functional activity or the diversity intra‐ and intercommunity, neither at phylum nor at family resolution; (iv) the functional activity and the ecological diversity of the bacterial communities are significantly affected by the body region, while the relative abundance is not. Obtained data confirm previous observations and provide new insight in the Forensic Veterinary field in terms of equally using them in order to derive a statistical model for the PMI estimation. As a future perspective, a contribution to the Forensic Entomology approach will be given in legal investigations when domestic or wild animals are involved, regardless of the presence of a hair layer.

## 1 INTRODUCTION

Microorganisms are the most abundant and genetically diverse organisms that inhabit all the natural Earth environments, including the most extreme ones, as well as other living organisms. Heterogeneous collections of microbes reside in and on different human body regions, especially skin, oral and genital mucosae, and the gastrointestinal tract. Studies carried out by the Human Microbiome Project Consortium (Consortium HMP, 2012) demonstrated that each individual owns a specific microbial community that can be used as a fingerprint for identification purposes, therefore, opening new frontiers in forensic research and investigations. In accordance with Locard's exchange principle, there is a precedent that microbes associated with human bodies have been accepted in the court of law as a type of nonhuman physical evidence with an evidentiary value during legal disputes. For example, in a context of physical violence, Borgula et al. (2003) demonstrated that bacteria could be recovered from a bite mark for at least 24 hr allowing to link the perpetrator to the victim. Hence, expanded use of microbes in forensic sciences can have a great potential.

Moreover, the communities of microorganisms, that inhabit human cadavers or animal carcasses after death, are investigated more and more often as a new tool for the estimation of the time since death by forensic scientists. After death, due to the collapse of the cardiovascular system and the breakdown of the immune system, microbes undergo an uncontrolled proliferation and they spread to body locations that were previously sterile. The postmortem transmigration of two bacterial species was also proved using a mouse model (Burcham et al., 2016). Microbes act as first body colonizers feeding on dead tissues and act as the main drivers of the decomposition process playing an important role in the recycling of the organic matter in the ecosystems (Benbow, Tomberlin, & Tarone, 2015).

The word “necrobiome” refers to a broader community of organisms including these postmortem microbial communities (Benbow, Lewis, Tomberlin, & Pechal, 2013). The terms “epinecrotic microbial communities” and “thanatomicrobiome” are used to describe those organisms residing and moving on the surface, respectively, (Pechal et al., 2014) or inside the organs of a decomposing body (Can, Javan, Pozhitkov, & Noble, 2014). Beyond the ecological role of microbes as main recyclers of the organic matter, their potential to be used as a new forensic tool for legal investigation purposes was promoted by the development of new microbiological techniques that allow the metabolic profiling of the microbial communities (Pechal et al., 2013; Weber & Legge, 2010) as well as by the advances in molecular biology, such as Next Generation Sequence (NGS) technologies. NGS allows the microbial DNA to be selectively processed in a very short time and at very competitive costs. Furthermore, NGS in association with newly developed bioinformatics tools enables the characterization of the environmental microbial communities through the identification of individual taxa of interest at species level including the noncultivable taxa. In this scenario, a new era of “Microbiome forensics,” as defined by Metcalf and coworkers (Metcalf et al., 2017), has started. Additionally, the authors showed how postmortem microbial communities can potentially be used as tool for time since death estimation, defining a “microbial clock” (Metcalf et al., 2013; Pechal et al., 2014). In fact, the bacterial communities seem to predictably change over time and space during the decomposition process in terms of structure and functional diversity (Metcalf et al., 2013; Pechal et al., 2014, 2013). Experimental trials also showed that within the dynamicity over time, these succession patterns are actually conserved among seasons (Carter, Metcalf, Bibat, & Knight, 2015). Despite some studies carried out on human bodies, mainly in the USA, the majority of the experiments on the postmortem microbiome has been performed using animals as models. The selection of an animal model depends on some factors including availability, cost, ease of handling, investigator familiarity, and anatomical/functional similarity to humans. In accordance to the latter factor, mainly swine carcasses, *Sus scrofa* Linnaeus, 1758, have been largely used (Carter et al., 2015; Pascual et al., 2017; Pechal et al., 2014, 2013). It is in fact well established that swines and pigs show a high anatomical and physiological similarity to humans and have the closest pattern decomposition pattern to them (Catts & Goff, 1992). Only in a few cases were mouse carcasses tested (Metcalf et al., 2013).

Despite the wide usage of animals as models in forensic studies, the effect of the fur on the decomposition process has so far never been explored. Compared to “Human Forensic,” the investigations of fundamental legal questions involving domesticated and nondomesticated animal species (“Veterinary Forensic”) has always had marginal attention and only recently, the interest in the discipline is increasing. This is due to five main factors as summarized by Cooper and Cooper (2008): (i) greater public concern about animal welfare; (ii) request of compensation or other legal recourses by the owners in cases of animal’s death, ill‐health, or injuries; (iii) wildlife protection; (iv) environmental forensics; and (v) control of animal‐derived products used for human and animal consumption.

The research presented here is based on the analysis and the comparison of decomposition, entomological, and microbiological data collected from domestic rabbits, *Oryctolagus cuniculus *Linnaeus, 1758. Rabbit carcasses with fur coat or shaved were left decomposing in order to provide information about the effect of the hair layer on the decomposer microbial communities. Unlike what most of the previous research has already focused on, this work applies principles of investigation relevant to human forensics on animals as the main subject in the Forensic Veterinary context. Reliable data were obtained and can be applied to both the Veterinary Forensic field and the broader field of comparative Forensic Medicine that covers areas of both human and veterinary interests (Cooper & Cooper, 2008).

## 2 MATERIALS AND METHODS

### 2.1 Experiment set up

Six rabbit carcasses (2.75–3.50 kg) (Table A1) were purchased from a pet food company (Kiezebrink, https://www.kiezebrink.co.uk/) and exposed on the roof of the School of Applied Sciences, University of Huddersfield, West Yorkshire, United Kingdom (53°38′36.5″N, 1°46′40.1″W). The experiment was set up in an urban area during spring/early summer time in 2015. Before being exposed, the dead rabbits, stored at −20°C for 3 days, were completely thawed and in thermal equilibrium with the ambient. Scissors and a common electrical trimmer were used to remove the fur coat from the torso of three rabbits. Carcasses with the same time since death (3 days) were chosen for the study in order to standardize the experiment as much as possible and avoid any potential microbiome variation due to a different starting PMI. All carcasses were placed on their right side on the top of approximately 4 cm of sterile sand in clean plastic containers (80 × 40 × 30 cm). Small holes were drilled into each side of the containers above the sand level in order to guarantee a continuous air flux and access for insects. The containers were covered with a lid in order to prevent access to birds and other small scavengers.

Using a digital scale with a precision of 50 g and a metal net (1 × 1 cm) placed under the carcasses, size and weight of the animals were recorded daily during the first week of the experiment and then once a week until the end of the experiment. In order to avoid any cross contamination, personal protection equipment (laboratory coat, safety laboratory goggles, sterile gloves, and facial masks) was worn by the operators during all the phases of the experiment set up and sampling.

### 2.2 Meteorological data collection

Average daily temperature and total rainfall data were obtained from the University weather monitoring station placed on the roof of the School of Applied Sciences, only a few meters away from the experimental area. Temperature data were later converted into Accumulated Degree Days (ADD, Day°C) by thermal summation models with a base temperature of 0°C, which accounts for temperature variation over decomposition (Megyesi, Nawrocki, & Haskell, 2005; Pechal et al., 2014). This measure is commonly used in forensic applications when extrapolations are made from experimental (i.e., lab) to field data of carrion decomposition with variable temperature (Pechal et al., 2014).

### 2.3 Insect collection and analyses

Diptera larvae and closed puparia were collected daily during the first week of the experiment and every 2 days for the second and third weeks; then twice a week for the fourth week and then once a week, until the last stage of decomposition. Some larvae and puparia were reared to adults to facilitate their identification. Adult flies were sampled using an entomological pooter or a hand‐net directly from the carcasses or from the experimental box. The European guidelines for forensic entomology were used to process the specimens. Morphological identification was performed on more than 200 adult flies and 100 larvae using an optical microscope Leica DME and a stereomicroscope Leica M60 equipped with a DFC425C camera. LAS (Leica) software was used to take measurements after manual calibration by the metric slide. Specific identification keys were used for the identification of the specimens (McAlpine et al., 1981; Merz, 1996; Porta, 1923–1934; Porta, 1949; Skidmore, 1985; Smith, 1986; Szpila, 2010). When necessary, the molecular approach was used to validate the morphological identification for specimens identified only at family level. QIAamp DNA Mini Kit (Qiagen) was used to extract total DNA from 10 adult specimens subjected to mechanical grinding using a plastic pestle. Larval tissues were obtained from three specimens following the protocol described in Tuccia, Giordani, and Vanin (2016). A partially modified protocol “DNA Purification from Tissue” (Qiagen) was used in order to increase the quality of the reaction by adding 4 μl of RNase A (4 mg/ml) (Promega, Madison, Wisconsin, USA) prior to ethanol precipitation step. Digestion by Proteinase K (100 µg/ml) (Promega) lasted 3 hr. Samples were left at room temperature for 5 min and incubated at 37°C for 30 min. RNase A was then inactivated at 70°C for 10 min. AL Buffer was added and the other steps were undertaken according to the manufacturer's instructions. The quality of each extraction was monitored using 1% w/v agarose/TBE gel electrophoresis. DNA was visualized after Midori Green Advanced (Geneflow Elmhurst, UK) staining. The COI barcode region (658 bp) was amplified using the universal primers LCO1490 and HCO2198 (Folmer, Black, Hoeh, Lutz, & Vrijenhoek, 1994). The GoTaq® Flexi Polymerase amplification protocol (Promega) was used as follows: 4 μl of Colorless GoTaqFlexi Buffer (5×), 2 μl of MgCl_2_ (25 mM), 0.5 μl of each primer (10 pmol/μl), 0.5 μl of Nucleotide Mix (10 mM), 0.25 μl GoTaq® DNA Polymerase (5 u/μl), 2 or 4 μl of DNA template, according to the quality of the extraction, and water to obtain a 20 μl final volume. The amplification program was set up on a BioRad C1000 Thermal Cycler (Bio‐Rad Laboratories, Inc.) as follows: 95°C for 10 min, 35 cycles of 95°C for 1 min, 49.8°C for 1 min, 72°C for 1 min followed by a final extension at 72°C for 10 min. Amplifications were confirmed by gel electrophoresis (1.5% w/v agarose/TBE) stained as described above. PCR products were purified using QIAquick PCR Purification Kit® (Qiagen) following the manufacturer's instructions. Purified amplicons were eluted in 40 μl of sterile/deionized water. Sequencing reactions were carried out by Eurofins Operon MWG—Ebersberg, Germany. The obtained FASTA sequences were compared with GenBank DNA sequences using BLAST‐n® (Altschul, Gish, Miller, Myers, & Lipman, 1990) and with home‐produced sequences as reported in Bortolini, Giordani, Tuccia, Maistrello, and Vanin (2018).

### 2.4 MICROBIOLOGICAL ANALYSIS

#### 2.4.1 Collection of bacterial samples

Bacterial swab samples for each carcass were taken from three body regions: oral cavity (internal region), skin (superior side exposed to the air environment), interface soil‐carrion (inferior side of the carcass leaning on the sand). Swab sampling was initiated the day of carcass exposure and was carried out weekly until the 68th day of the experiment when the dry stage of decomposition began. The same procedure was followed for carcasses with and without fur. In the first case, swabs were rubbed both on the fur surface but also touching the underlying skin. All the oral cavity around and the same sized area on the exposed skin and under the carcasses were swabbed for 30 s using dry DNA‐free sterile cotton swabs (Standard Swab Kit, Scenesafe™).

For each body region, triplicate samples were collected and immediately stored in ice. One set was used the same day of the collection in order to assess the phenotype of the microbial communities by BIOLOG EcoPlates™ as described below and in Pechal et al. (2013). The two remaining swabs were stored at −20°C until further molecular processing such as DNA extraction for 16S rRNA gene amplification (Table A2). Per each decomposition stage, DNA was isolated from selected samples after the elaboration of functional data as explained below.

#### 2.4.2 Functional characterization of the postmortem microbial communities

BIOLOG Ecoplates™ were used to investigate the phenotype of the microbial communities sampled as a function of their ability in using different carbon sources. In this study, the following protocol was carried out according to the manufacturer's instructions (Weber & Legge, 2010). Every single swab was gently shaken in 15 ml of 0.9% NaCl sterile solution to obtain a microbial suspension. One hundred and fifty microliters of suspension were inoculated directly into each of the 96 wells of a BIOLOG EcoPlate™. Eighteen array plates were prepared each time. The absorbance of each well was measured automatically at 590 nm every 24 hr for 5 days (120 hr) at a constant temperature of 25°C using a SPECTRO Star NANO (BMG LABTECH) spectrophotometer. Positive plates defined by color changing were noted and the microbial functionality index was calculated as the ratio between the numbers of positive wells on the total 31 carbon sources. The obtained value expressed as percentage varied from 0% to 100% with 0% being the lowest diversity and 100% being the highest one. The variation of results within samples was calculated as 100 × *i*/31, where *i* is the number of carbon sources showing differences in the triplicates, that is, not all positive or all negative (Mulchay and Edenbord, 2007).

#### 2.4.3 Molecular characterization of the postmortem microbiome

Three groups of six swabs were chosen for molecular analyses, in order to cover the entire duration of the decomposition process, and to analyze the bacterial community associated with the decomposition and not with the animal when alive. Each group referred to active decay, advanced decay, and dry stage, respectively. In turn, two types of carcasses (with = F and without fur = NF) were chosen for each group for the purposes described above. For each carcass, three body regions’ samples were selected based on the EcoPlate metabolic profiles in order to evaluate the potential different distribution of the microbial population (Table A2).

##### 2.4.3.1 DNA extraction

Fast Stool DNA Mini Kit (Qiagen) was used to extract total bacterial DNA from swabs using the manufacturer's protocol. The extremity of each swab was cut after swabbing on the selected body regions and put in a 2 ml collection tube and stored in ice. One milliliter of InhibitEX Buffer was added and each suspension was left at room temperature for 4 hr, partially modifying the manufacturer's instructions, and mixed every 60 min to improve the quality of the cellular suspension. All following steps were performed according to the manufacturer's instructions.

##### 2.4.3.2 Generation of sequencing library and 454‐pyrosequencing

DNA amplification was carried out on the V1‐3 region of the 16S rRNA gene, widely used as a molecular marker. Universal modified primers Gray28 FW (5′‐TTTGATCNTGGCTCAG‐3′) and Gray519 RV (5′‐GTNTTACNGCGGCKGCTG‐3′) were used as described by Pechal et al. (2014). HotStarTaq Master Mix (Qiagen) was used to prepare a master reaction of 50 μl for each DNA template: 25 μl of a ready‐to‐use mixture containing HotStarTaq DNA Polymerase (5 u/μl), Qiagen PCR Buffer, and dNTPs; 2 μl of each primer (10 pmol/μl); 10 μl of total extracted DNA and 11 μl of H_2_O RNase free. The amplification program was set on a BioRad C1000 Thermal Cycler (Bio‐Rad Laboratories, Inc.) as follows: initial heat activation at 95°C for 15 min; denaturation at 94°C for 1 min; annealing at 58°C for 1 min; elongation step at 72°C for 1 min; and a final extension at 72°C for 10 min. Thirty‐five cycles were given as input. The quality of the amplification reactions was confirmed by gel electrophoresis (1.5% w/v agarose/TBE). Amplicons were purified using QIAquick PCR Purification Kit® (Qiagen) following the manufacturer's instructions and eluted in 30 μl of Elution Buffer (Tris‐buffer/EDTA). Quantification of purified DNA was performed using the Quant‐iT dsDNA HS Assay kit on a Qubit 3.0 fluorimeter (Thermo Fisher Scientific, Waltham, Massachusetts, USA). NGS—Laboratory of Eurofins Genomic Operon (Germany) performed GS‐Junior Pyrosequencing reactions with Titanium Series as part of the 454‐Roche sequencing system.

#### 2.4.4 Community and biostatistical data analysis

The 454 reads were converted into FASTA sequences with the 454 tool sffinfo [sffinfo ‐s file.sff > file.fasta] and filtered by quality criteria as by default settings in QIIME v1.6.0 (Caporaso et al., 2010). Chimeric sequences were sorted out as “no relative” and not taken into account for subsequent analysis. The Operational Taxonomic Units (OTUs) were defined on a 98% identity threshold, and classified according to the SILVA 16S reference taxonomy for Bacteria (SSU RefNR 123 dataset), as reported in http://www.arb-silva.de/projects/ssu-ref-nr/ (Data Analysis Service for Ribosomal DNA reads, Eurofins Genomics, Ebersberg, Germany). The composition of bacterial taxa was summarized as the ratio of the number of read OTUs to the number of reads for all samples. Phylum and family taxonomic resolutions were separately investigated and taxa with an abundance <2% of the total read OTUs were classified as “rare.” The α‐diversity metrics were calculated using QIIME version 1.6.0. The species richness was investigated through rarefaction analysis and the Chao1 estimator was applied to evaluate the asymptotic species richness. The richness and the evenness within each sample were expressed as Shannon (H′) and Gini–Simpson (1/D) diversity indexes.

PAST v. 3.20 software (Hammer, Harper, & Ryan, 2001) was used applying the Bray–Curtis similarity index following Pechal et al. (2013) to run the permutational multivariate analysis of variance (PERMANOVA) and the principal component analysis (PCA) (e.g., β‐diversity evaluation).

Carrion conditions (the presence or absence of fur) were separately analyzed in order to better describe their effects on the distribution of the microbial communities during the decomposition process (Pechal et al., 2013). “NF” and “F” are used in the text to refer to the absence or presence of the fur on animals, respectively. Descriptive values are reported as average ± standard deviation.

## 3 RESULTS

### 3.1 Meteorological data and body mass loss

**Figure 1:**
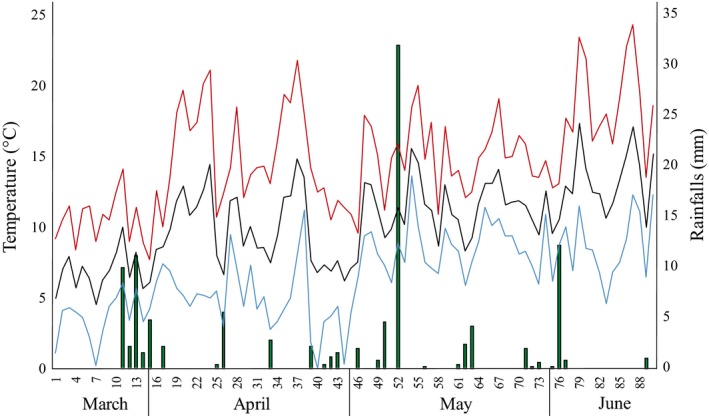
Meteorological data (March–June 2015). Average (black line), minimum (blue line), and maximum (red line) temperature and amount of precipitations (mm, green histograms) occurring between March and June 2015. Numbers below X axis refer to days of the experimental trial

In order to determine how biological data are influenced by environmental contexts, the monitoring of meteorological data including temperature, humidity, rainfall, and wind is essential when decomposition studies are performed. Over the experimental period, March–June 2015, the average temperature was +10.4 ± 2.9°C (Figure 1). Maximum temperature was +24.3°C and the minimum temperature was +0.2°C. Average precipitation was 1.2 ± 4.0 mm (Figure 1).

**Figure 2:**
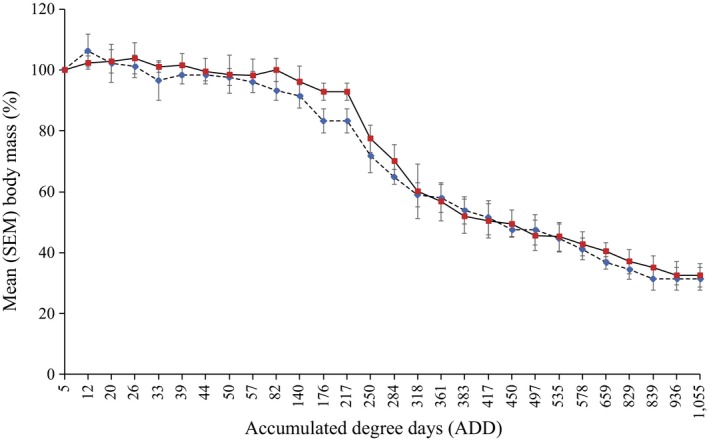
Average weight of three carcasses with fur (red line) and without fur (blue line) through time expressed as Accumulated Degree Days (ADD) calculated by thermal summation models with a base temperature of 0°C

A progressive loss in carrion mass was observed during the decomposition process (Figure 2). After an initial stable phase occurring after death, the mass of the carcasses decreased very slowly. After 3 weeks (164 ADD) it was still around 90% of the initial mass. A further reduction down to 50% occurred only around 383 ADD during this period corresponding to the advanced decay stage. The decreasing in body mass slowed down in a linear way and the stabilization of the average weight value occurred at the beginning of June (839 ADD) when carcasses entered the dry stage.

### 3.2 Entomological findings

**Figure 3:**
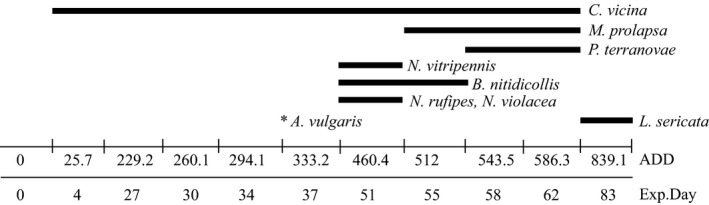
Insect species temporal distribution. Distribution of the insect species observed over the experiment period. Time is expressed as Accumulated Degree Days (ADD) and as experimental days where 0 is the day of the exposure of the carcasses. No differences were detected between F and NF carcasses

The analysis of the entomofauna, which plays an important role in reducing carcass mass, is a key factor in order to better understand the decomposition phenomena since necrophagous insects influence and interact with the microbial communities (Crippen, Benbow, & Pechal, 2015). Species belonging to Calliphoridae, Muscidae, Piophilidae, and Sciaridae families were collected and identified with no differences in their arrival and presence in the F and NF carcasses. Within Calliphoridae *Calliphora vicina*, Robineau‐Desvoidy, 1830 was the most abundant species followed by *Protophormia terraenovae*, Robineau‐Desvoidy, 1830. A single specimen of *Allopiophila vulgaris* Fallen, 1820 and one of *Lucilia sericata* (Meigen, 1826) were collected at the beginning and late in the decomposition process respectively. Only a few specimens of *Necrobia violacea* (Linnaeus, 1758) and *Necrobia rufipes* (De Geer, 1775) (Coleoptera: Cleridae) were observed and collected during the dry stage. All species found were necrophagous, feeding on human cadavers or animal carcasses, with the exception of *Nasonia vitripennis* (Walker, 1836) (Hymenoptera: Pteromelidae), a parasitic wasp emerged from Diptera puparia. A complete list of the entomofauna and a timeline of its appearance on the carcasses are summarized in Figure 3.

### 3.3 Microbial functional activity

The functional activity of microbial communities developed on rabbit carcasses was evaluated by metabolic fingerprinting of each analyzed body area through the decomposition process.

#### 3.3.1 Oral cavity

**Figure 4:**
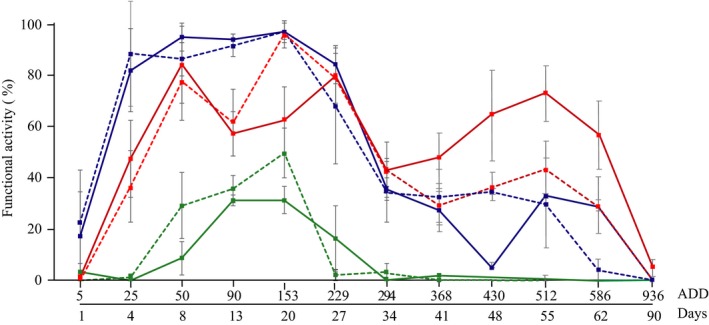
Average microbial communities’ functional metabolic activity (%) observed per each body region over time Accumulated Degree Days (ADD, Days). Decomposition stages are reported according to carcasses observation. Blue lines refer to oral cavity samples, red lines refer to soil interface samples, and green lines refer to skin samples. Each line represents the average value derived by three carcasses: F condition is labeled as full line, NF condition is labeled as dashed line. The average functional variation (%) is reported as error bar

The average value of the functional activity observed on the day of the first sampling was 22.6% and increased to 88.2% 3 days later (25 ADD) when the bloat stage occurred. During the active decay, high values were still observed reaching the highest peak at 96.8% (Figure 4). A reduction in the microbial functional activity was observed when the carcasses entered the advanced decay stage (180 ADD). In the late advanced decay, the functional activity showed constant values between 30.1% and 34.1%. A final strong decrease in the activity of the microbial community of the mouth was recorded when the dry stage occurred, about two months after the beginning of the experiment (512 ADD). The average values of the functional activity calculated for carcasses with and without fur were very similar exhibiting the same trend over all the decomposition process with the exception for values recorded from 430 ADD (Figure 4).

#### 3.3.2 Skin

Compared to the previous area of the body, microbial activity on skin samples was delayed and only detected a few days after the carcasses’ exposure. The average values recorded at this stage differed slightly between F and NF rabbits (10% vs. 30%). The highest values were reached during the active decay, 30% and 45%, until an evident and drastic decrease was observed in carcasses NF (153 ADD) (Figure 4). On the contrary, the functional activity drop was slightly smoother in carrion F giving the curve the typical belt shape, and, in less than one week after the maximum peak of activity, the functional activity reach values below 10% in both cases. Over the rest of the decomposition process no activity was detected except for a weak signal recorded during the dry stage, likely due to a strong rain event which occurred around the 50th day of the experiment (430–512 ADD) (Figure 1).

#### 3.3.3 Interface soil‐carrion

Microbial activity was observed a few days after carcasses exposure as the bloat stage occurred (25 ADD). Activity increased to 77.4% at the beginning of the active decay phase reaching later (153 ADD) the maximum values, over of 80 and 95.7% for carcasses F and NF, respectively (Figure 4). As the advanced decay occurred, a general weak decrease was observed. At 229 ADD, the detected activity was lower than 80% and values around 40% were reached at 294 ADD. An increased peak of activity could be observed, which was remarkable as it occurred despite the carcasses having already lost most of their water content in conjunction with rainfalls occurred between 430 and 512 ADD (Figure 1), as previously mentioned for the skin samples. From 512 ADD, a decreasing trend was observed up to values <10% (Figure 4).

PERMANOVA revealed that the mean functional activity of the microbial communities significantly changed over time (*p* = 0.0005), but was not significantly affected by the presence of the fur (*p* = 0.963). In contrast, the body region was found to provide a significant contribution in modulating the metabolic activity of the postmortem microbiome (*p* = 0.000). The interaction between the variables was not significant under any circumstances.

### 3.4 Necrobial community composition

In order to characterize the postmortem microbiome, pyrosequencing analysis of the V1‐3 region of the bacterial 16S rRNA gene was successfully performed on all 18 samples producing a total of 79,082 DNA sequences classified as OTUs based on the SILVA rRNA database. Eighty‐five bacterial families were identified within 14 phyla.

**Figure 5:**
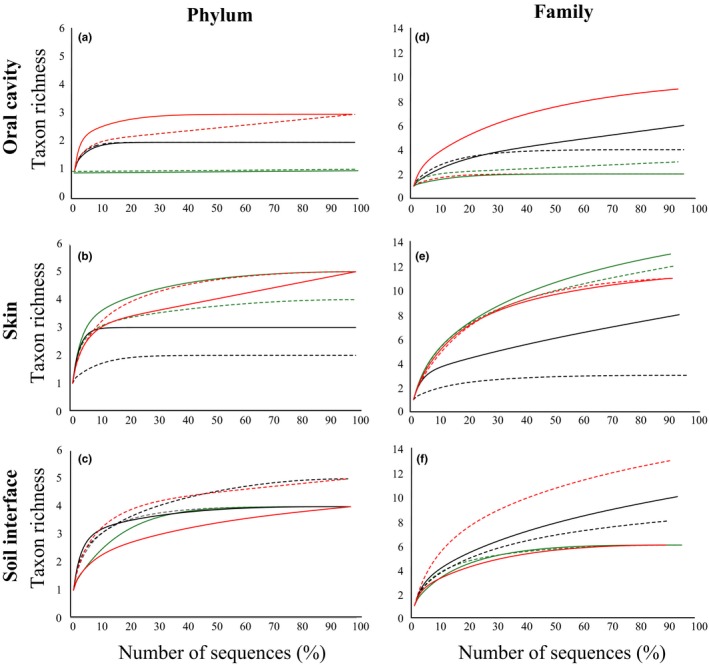
Rarefaction plots of phyla (%) and family richness. Rarefaction curves are shown for phylum and family taxa combined per body region (a) and (d) oral cavity, (b) and (e) skin, (c) and (f) Soil interface. Each line color refers to the three sampling points: green lines are for 50.1 Accumulated Degree Days (ADD) (March), black lines are for 294.1 ADD (April), and red lines are for 586.3 ADD (May). Continuous lines = F, dashed lines = NF. The number of DNA sequences Operational Taxonomic Units (OTUs) is expressed as percentage

Rarefaction analysis showed that asymptotes were reached more rapidly in almost all the samples when the phylum is taken into account. Exceptions refer to carrion samples NF oral cavity 586 ADD, NF skin 586 ADD, NF, and F soil interface 586 ADD (Figure 5a–c). On the contrary, when microbial families were considered as referring taxa, only six samples out of 18 were sufficiently sequenced in order to observe all taxa: F oral cavity 50 ADD, NF oral cavity 294 ADD, NF skin 294 ADD, F and NF soil interface 50 ADD, and F soil interface 586 ADD (Figure 5d–f).

At the phylum level, Chao‐1 index, the richness measure, was significantly affected by the sampling region (*p* = 0.026) and by the presence/absence of fur (*p* = 0.039). The same index calculated at the family level did not show any significant difference between the presence and absence of fur and the investigated body regions (*p* = 0.118 and 0.057, respectively). The richness of the bacterial communities did not show any statistically significant change neither over time at both phylum and family level (*p* = 0.350 and 0.082 respectively).

### 3.5 Taxa relative abundance—Phylum

**Figure 6:**
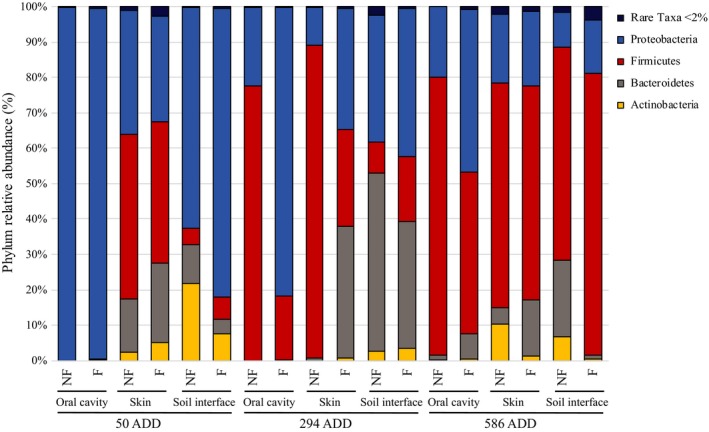
Bacterial communities’ relative abundance at Phylum level. Bacterial composition is expressed as % values based on the total DNA sequence read and clustered per each Phylum: Proteobacteria (blue), Firmicutes (red), Bacteroidetes (gray), and Actinobacteria (yellow). The cut‐off per rare taxa (dark blue) was set as <2%. For each point of samplings expressed in Accumulated Degree Days (ADD), histograms are clustered per body region and per each body region, data referring to “no fur” or “fur” conditions are named as “NF” and “F”, respectively

The analyses performed at phylum level showed that four main phyla made up the postmortem microbiome after death. Early, during the initial decomposition process, Proteobacteria was the most abundant phylum (70.7%), followed by Firmicutes (14.4%), Bacteroidetes (8.0%), and Actinobacteria (5.9%) (Figure 6). Over decomposition, Proteobacteria progressively decreased (38.5% at 294 ADD and 21.9% at 586 ADD) becoming the second most abundant phylum. In contrast, Firmicutes progressively increased becoming the most abundant phylum during the middle and late stage of the decomposition (40.5% at 294 ADD and 64.6% at 586 ADD). Bacteroidetes slightly increased at 294 ADD (19.2%) and then became less abundant during the last stage (8.5%). The percentage of Actinobacteria was consistent throughout. All the other minor taxa were clustered as “rare taxa” each one of them representing less than 2% of the total.

#### 3.5.1 Oral cavity

In the oral cavity, Proteobacteria was the dominant taxon representing 99.4% of the total at the beginning of the active decay stage (50 ADD) in both F and NF samples. During the advanced decay (day 34, 294 ADD) Proteobacteria decreased to 81.6% but it was the most abundant phylum in NF samples, followed by Firmicutes (18%). In contrast, Proteobacteria showed a significant reduction to 21.9% in F communities and Firmicutes became the most abundant taxon (77.7%). However, in the dry stage (day 62, 586 ADD), Firmicutes was still the most abundant in the NF communities (78.4%), followed by Proteobacteria (20%). While in F samples, Firmicutes and Proteobacteria were the most abundant taxa (45.5 and 46.2% respectively), followed by Bacteroidetes (7.2%) and Actinobacteria (0.7%) (Figure 5).

#### 3.5.2 Skin

The first sampling of the skin (day 9, 50 ADD) showed a different microbial pattern compared to the oral cavity. In fact all the main phyla were detected, but no significant differences were observed depending on the presence or absence of the fur. Firmicutes represented the most abundant taxon in NF and F (46.4 and 39.9% respectively) followed by Proteobacteria (35.2% and 29.9%). Bacteroidetes and Actinobacteria were less abundant. Bacteroidetes represented 15.1% and 22.1% of the total in NF and F, while Actinobacteria were present as 2.4% and 5%. During the advanced decay stage (day 43, 294 ADD), Firmicutes were the most abundant taxon in NF skin sample (88.5%) followed by Proteobacteria which was up to 10.4%. On the contrary, Firmicutes were three times less abundant in F rabbits representing the third most abundant phylum after Bacteroidetes (37.1%) and Proteobacteria (34.3%). Actinobacteria were detected only up to 0.8%. Firmicutes showed the highest percentages among all the other phyla in both types of animals until the last samples (63.6% NF, 60.5% F). Equally, Proteobacteria showed a very similar distribution pattern related to the presence of the fur (19.2% NF, 22.2% F). Bacteroidetes and Actinobacteria were the least abundant taxa as observed during the first sampling and still, a slight difference was observed between F and NF animals.

#### 3.5.3 Soil interface

As observed for skin samples, the communities of the soil–carcass interface were represented by all the four main phyla.

Proteobacteria was the dominant phylum during active decay (day 9, 50 ADD) representing 61.4% and 81.7%, in NF and F, respectively, and progressively decreased in the late and final stage of the decomposition (35.8% in NF and 41.8% in F at 294 ADD, 9.9% in NF and 15.1% in F at 586 ADD). On the contrary, an increment of the Firmicutes was observed over the time. From 4.7% NF and 6.2% F initially detected (50 ADD), values increased up to 8.8% NF and 18.3% F in the advanced stage (294 ADD) and finally reached 60.2% NF and 79.2% F during the dry stage. Bacteroidetes initially were present at 10.9% NF and 6.2% F and then followed a bell shaped trend over the three sampling points. In fact, after increasing to 50.2% NF and 36.1% F (294 ADD), the distribution values reduced to 21.6% NF and 1.1% F at 586 ADD. Actinobacteria were present in the highest fraction within soil samples in the early stage of the decomposition (21.8% in NF and 7.7% in F, 50 ADD) and turned down later in the decay (2.75% NF and 3.3% F, 294 ADD). Low values were even recorded at 586 ADD (6.7% NF, 0.4% F). Still, no significant difference was observed depending on the presence of the fur.

### 3.6 Taxa relative abundance—Family

**Figure 7:**
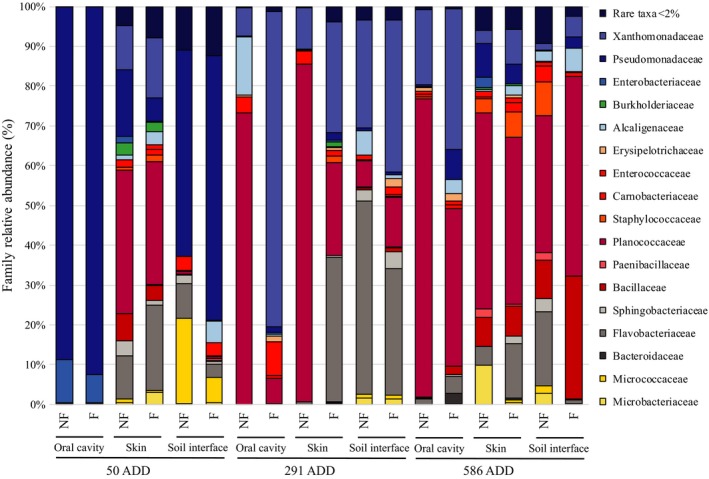
Bacterial communities’ relative abundance at Family level. Bacterial composition is expressed as % values based on the total DNA sequence read and clustered per each bacterial Family. 17 taxonomic families were identified with a cut‐off >2%. The others were named as “rare taxa.” For each point of samplings expressed in Accumulated Degree Days (ADD), histograms are clustered per body region and per each body region, data referring to “no fur” or “fur” conditions are respectively named as “NF” and “F”

The screening of the DNA readings allowed the identification of 85 bacterial families based on the SSU RefNR 123 dataset, but only 17 taxa with a frequency >2% were taken into account for further statistical analyses. In the early phase of the decomposition process, Pseudomonadaceae were distributed predominantly in the oral cavity and at the soil–carcass interface, while Planococcaceae dominated the skin from the beginning to the end of the experiment. Also, the latter represented the most abundant taxa at the very last stage and in every analyzed body region. From the middle of the process, Pseudomonadaceae were replaced by Xanthomonadaceae, Flavobacteriaceae, and Bacillaceae, which became considerably abundant at the interface level between soil and carcasses at the end of decomposition (Figure 7).

### 3.7 Ecological analyses of the bacterial communities

**Figure 8:**
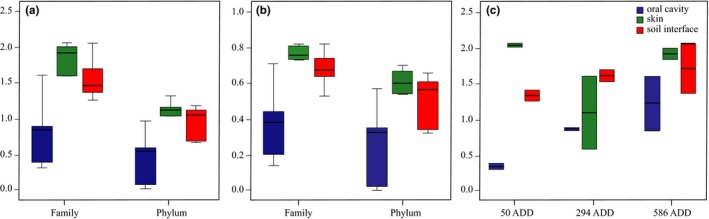
Diversity indexes. Shannon (a) and Simpson (b) diversity indexes. Box plots are clustered based on Phylum and Family values. (c) Diversity within families increases over time in oral cavity and soil interface samples while an inverted bell shape was observed in skin microbial communities’ diversity. Different colors represent the three body regions: oral cavity (blue), skin (green), and soil interface (red)

The α‐diversity of each community was measured calculating the Shannon–Wiener (*H*′) and Gini‐Simpson (1/*D*) indexes. The bacterial communities in the oral cavity showed a lower diversity compared to skin and soil‐interface samples (Figure 8). The family diversity within the oral cavity and soil–interface samples increased over the decomposition process, while an inverted bell shape was observed for skin samples (Figure 8c). PERMANOVA showed significant differences between the body regions for both indexes at the family level (*H*′ *p* = 0.023; 1/*D* *p* = 0.028), whereas only *H*′ were significantly different at the phylum level (*p* = 0.006) being 1/*D* not significantly different (*p* = 0.153) demonstrating that the diversity of the bacterial community was in general affected by the body region. In contrast, all the p‐values obtained comparing the presence and absence of fur were >0.05 (*H*′ Phylum *p* = 0.924, *H*′ Family *p* = 0.609, 1/*D* Phylum *p* = 0.932, 1/*D* Family *p* = 0.691) demonstrating a nonsignificant effect of the presence/absence of fur on the bacterial community general diversity. The variable time did not show any significant difference in the analysis (*p* > 0.064).

**Figure 9:**
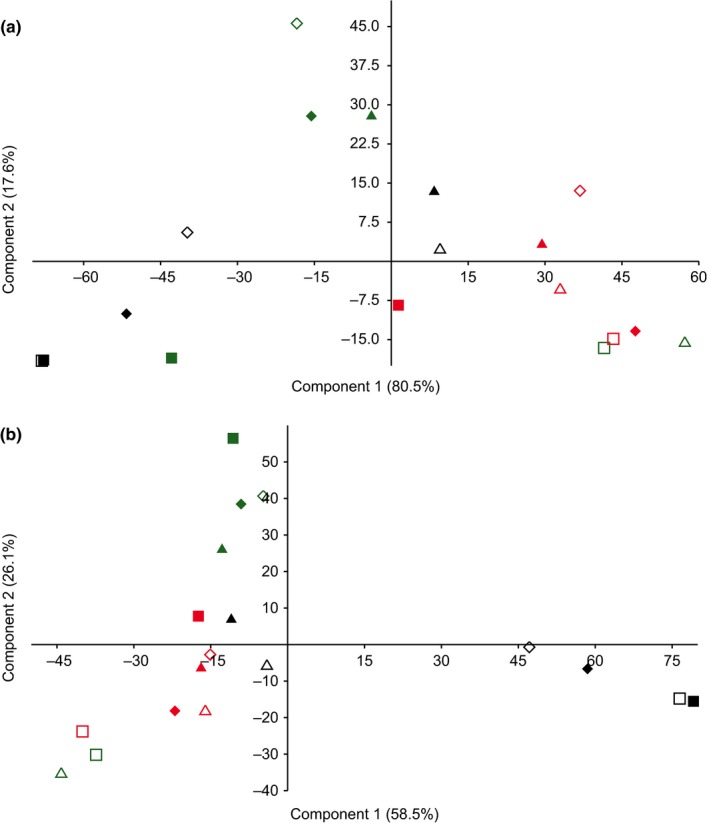
*β*‐Diversity of the microbial communities. Principal Component Analysis (PCA) based on data referring to phyla (a) and families (b). A color code has been assigned to the time of the sampling: black (March‐50 Accumulated Degree Days [ADD]‐active decay), green (April‐294 ADD‐advanced decay), and red (May‐586 ADD‐dry stage). A shape code defines the body regions: squares define the oral cavity, triangles define the skin, and the diamonds represent the interface between soil and carrions. Empty or fill shapes respectively refer to NF or F condition

The intercommunity comparison assessed by PCA (Figure 9) showed that “time” is the variable which most significantly affects the separation of the bacterial communities at both taxonomic levels, phylum (*p* = 0.013) and family (*p* = 0.001). On the contrary, no significant effect depending on either the body region or the fur presence at any level of resolution was observed (Body region—Phylum *p* = 0.127) (Body region—Family *p* = 0.052) (Fur—Phylum *p* = 0.391) (Fur—Family *p* = 0.576).

In summary, the characterization of the postmortem microbial communities developed on rabbit carcasses performed at functional and molecular level and supported by statistical analysis revealed that: (i) distinct and significant temporal changes in terms of metabolic activity and taxa distribution can be tracked throughout the decomposition process; (ii) the richness and the diversity of the bacterial communities does not vary significantly over time, however, species (OTUs) do not change; (iii) the presence/absence of the fur on the carcasses does not significantly affect either the bacterial communities functional activity or the diversity intra‐ and intercommunity, both at phylum and at family resolution; (iv) the functional activity and the ecological diversity of the bacterial communities are significantly affected by the body region while the relative abundance is not.

## 4 DISCUSSION

In the last decade several research groups have conducted studies to better understand the decomposition process and the interaction between abiotic and biotic factors, for example, microbes and insects (for a review see Benbow et al., 2015). The interest in this topic actually goes beyond a solely ecological perspective, as it focuses on the decaying of bodies, both human cadavers and animal carrions, in order to obtain reliable data for forensic investigations. Since 1980, students and faculty members at the University of Tennessee have conducted experimental studies of the natural decay process in humans. Before this time, rats and pig carcasses were tested as model organisms for humans. The observations derived that time, and largely confirmed at present, lead to describe the decomposition as a dynamic ecological process characterized by several physical, chemical, and biochemical changes. These initial microscopic changes finally cause a macroscopic transformation of cadavers and carcasses, which rehabit the ecosystem as resource subsidies having a huge impact on it (Tomberlin, Mohr, Benbow, Tarone, & VanLaerhoven, 2011). These changes are the results of the action and interactions between biotic and abiotic factors dependent on environmental conditions (temperature, humidity). In fact, it is well established that temperature and humidity play a direct role on the decay process, as well as an indirect one on the biome developing on the carrion/cadaver composed mainly by insects and microbes, and on vertebrate scavengers (Smith, 1986). The species distribution and competitive ability of all these living organisms correlate with habitat modifications and production of specific conditions inferred by the above mentioned abiotic variables (Tomberlin et al., 2011). As the decomposition moves forward through several stages a progressive reduction of the body mass is observed. This is in part due to the removal of the flesh by insects and other scavengers whether they have access to the body, but it is also associated with a loss in the water content. The latter, in turn, is the *conditio sine qua non* for the development and survival of living organisms. In fact, in dry environmental conditions, biotic activity is dramatically reduced and the whole process is slowed down.

In this work, an initial stationary phase lasting for 12 days occurred after rabbit carcasses placement (82 ADD), likely due to the relatively low temperature, as typical of the spring season in the United Kingdom. The carcasses then lost weight as the decaying process progressed. The skeletonized phase was not reached under any circumstance and no significant differences were observed between carcasses with fur and without fur. Given the environmental conditions and the small size of the rabbit carcasses, the removal of the biomass (decomposition rate) was quite rapid if compared to bigger animals at the same conditions. Previous studies performed on small carcasses, like mice (*Mus musculus* Linnaeus, 1758) (Metcalf et al., 2013), lizards (*Iguana iguana* Linnaeus, 1758, and *Ctenosaura similis *Gray, 1831), and toads (*Bufo marinus *(Linnaeus, 1758)) (Cornaby, 1974), showed that smaller the amount of the available flesh, the faster the decomposition. This negative correlation between size and decomposition rate was also reported in a comparison study between adult and infant human remains and a pig model showing that the amount of the initial matter affects how fast a corpse/carcass is consumed, rather than the species (Micozzi, 1986). In the absence of vertebrate scavengers (e.g., birds, wolfs, foxes, dogs, etc.) the necrophagous entomofauna represents the main biotic factor taking part to the physical consumption of carrion. According to the “semiochemical hypothesis” by Davis and coworkers, insect species are attracted on carrion by microbial volatile organic compounds released as products of the microbial metabolism which is involved in the breakdown of the soft tissues (Davis, Crippen, Hofstetter, & Tomberlin, 2013). In addition to the chemical composition of the pabulum, the water content is an essential condition for the deposition of the eggs and larval development, confirming the existence of a physical interaction between biotic and abiotic factors. Most of the fly species collected from the rabbits reflect what is already known about the seasonal distribution of carrion‐breeding species in the United Kingdom. *Calliphora vicina* and *P. terraenovae, *commonly known as spring blow flies species, were in fact the most abundant colonizers of the carcasses over the entire period of the experiment and over the middle–late phase, respectively. The small size of the carcasses and the meteorological conditions also explain the low number of beetles (*N. violacea* and *N. rufipes*). Furthermore, the lack of other beetle species is potentially related to the consumption of the majority of the tissues by the fly maggots. The parasitoid wasp (*N. vitripennis*) is associated with the presence of their host, fly pupae, and does not play any role in the decomposition process. No differences in the pattern of insects feeding on rabbits were observed based on the presence of the fur on the animals, reasonably due to the fact that the first eggs deposition occurs at the level of the oral cavity and other orifices, which were equally treated and exposed in this experiment.

Further evidence of the role of abiotic factors in driving the whole decomposition process derived from the postmortem bacterial communities’ metabolic activity detected on the carcasses. Within the monitored temporal range, between time 0 (= 5 ADD) and 936 ADD, the average microbial functional activity showed a bell‐shaped distribution at least at the oral cavity and skin level (Figure 4). At 512 ADD, when all the carcasses had already entered the dry stage, these two body regions showed a stationary phase of activity. On the contrary, at the carcass–soil interface, a second peak of activity occurred. When comparing to the meteorological records (Figure 1) between day 41 and 52, *alias *368 ADD, and 471 ADD, precipitation occurred and reached 32 mm on day 52, the highest value over the experimental time. The observed reactivation peak is likely due to the rain as an environmental perturbation which acted on the already decomposed carcasses as a new *stimulus/*activator for the microbial community metabolism, as also described by Forbes and Carter (2016). Furthermore, these data can be better elucidated by the experimental design: the carcasses were located inside plastic boxes equipped of lateral holes for gas exchange and insects arrivals, but the lid prevented the direct rain on the carrion. However, the same days were also characterized by high wind speeds (the average wind speed was of 14.4 km/h between day 41 and day 52 with 23.4 km/h being the maximum reached on day 52) so that the soil first got easily wet and the optimal microbial grow conditions were restored.

From our analyses, it emerges that the microbial fauna associated with the oral cavity and the skin of the carrions during the first phases of the decomposition is separated by the rest of the communities suggesting the major contribution of the carrion itself rather than the environment. The environment, in fact, seems to play a role in modulating the communities in the late phase of the process. In fact, soon after death, the postmortem microbial community is mainly endogenous, that is, it is typical of the preexisting living organism and it is related to the environment, life, and dietary habits (Crippen et al., 2015). The following purge of tissues as a consequence of the cellular autolysis causes an increase in the availability of food sources which bring about a rapid proliferation of the “preexisting” taxa and, in turn, allow the development of exogenous taxa from the environment. The “local” microbial pool is even enriched and might be subjected to perturbations by the arrival of the necrophagous flies which, carrying their own microbial pool, have important effect on microbial community assembly during the decomposition (Crippen et al., 2015; Pechal et al., 2013; Weatherbee, Pechal, & Benbow, 2017). For example, flies acquire bacteria from the environment and disperse them on corpses via dislodgment from the exoskeleton, fecal deposition, or regurgitation of internalized bacteria (Crippen et al., 2015). So far, the complexity of this ecological system increases and changes occur among life kingdoms. The copresence of these competitive biotic factors finally resulted in a depletion of carbon sources and water content which is related with the beginning of the dry stage with a consequent reduction in microbes’ functional activity, which occurred here at around 153 ADD (Figure 4).

In this study, the key role of water as the main abiotic driver of the microbial decomposition process was inferred by the wetness/air‐exposure of the analyzed body regions. The wettest anatomical areas, such as the oral cavity and the right lateral region leaning on the soil, likely promoted the development of bacterial communities that were observed to be constantly active until the body entered the advance and dry stage. At this point, a progressive decrease in the microbial functional activity was detected with a similar pattern shown by the oral cavity and the lateral region.

On the contrary, the postmortem microbial community colonizing the skin showed a scarce functional activity mainly due to the high dry nature of this body region. The skin dryness in fact negatively affected the development of the microbial communities over the whole experiment. The same remarkable difference in terms of functional activity across those two body regions came out from Pechal and coworkers’ experiment carried out on swine carcasses (*S. scrofa*) (Pechal et al., 2013). Strong support of these observations is provided by the statistical analysis of our study, which confirm the significant effect of the variable “time” and “body region” in modulating the microbial functional activity.

The taxa characterization within the postmortem microbial communities revealed four main phyla including the gram‐negative Proteobacteria and Bacteroidetes and the gram‐positive Firmicutes and Actinobacteria. These findings confirm several previous postmortem microbiological studies performed on animal carrion such as pigs (*S. scrofa*) placed both on the ground (Pechal et al., 2014, 2013; Weatherbee et al., 2017) or in fresh water, salmon (*Oncorhynchus keta* (Walbaum, 1792)) (Pechal & Benbow, 2016), and on human cadavers (Hyde, Haarmann, Petrosino, Lynne, & Bucheli, 2015). In the latter case, analyses focused both on external anatomical areas and internal organs (Can et al., 2014). Our results clearly demonstrated that bacterial communities are temporally distinguishable and that “time” as “decomposition progression” is the variable which most causes changes in the bacterial communities’ relative abundance (Figures 6, 7 and 9). Despite the difference in monitored time lapses, the trend relative abundance of taxa in our work is in agreement to what has been already published at least for Proteobacteria and Firmicutes (Hyde et al., 2015; Pechal et al., 2014, 2013; Weatherbee et al., 2017).

We observed that the Proteobacteria phylum is the most abundant in the early phases and decreases as the time passed by, while Firmicutes increase over time (Hyde et al., 2015; Pechal et al., 2014, 2013). Within Proteobacteria, Pseudomonadaceae represented the highest fraction in the active decay stage while Xanthomonadaceae was the major taxon during the late stage of the decomposition. In particular, the increase in Xanthomonadaceae over time on skin and grave soil was already observed by using mouse and swine carcasses (Metcalf et al., 2013; Pascual et al., 2017). This datum, as in accordance with what Pechal observed on pig carcasses (Pechal et al., 2014), strengthens the hypothesis that the Xanthomonadaceae family may be a key contributor to the general process of the decomposition regardless the host (Hyde et al., 2015). Within Firmicutes, Planococcaceae represent the highest fraction and were previously reported in association with vertebrate carcasses (Metcalf, Carter, & Knight, 2015; Pechal & Benbow, 2016). *Alcalignes* and *Ignatzschineria* genera were predominant in Proteobacteria: Xanthomonadaceae and the latter were previously reported as dominant genus usually associated with flies belonging to the Sarcophagidae family. The major number of OTUs within Actinobacteria: Microbacteriaceae were assigned to *Leucobacter* and *Arthrobacter *genera while within Bacteroidetes: Flavobacteriaceae most of the OTUs were assigned to *Flavobacterium *and *Myroides*. In addition to temporal changes of the mentioned taxa, richness, diversity indexes, and functional activity were diverse in different body regions, but not the relative abundance of the taxa. In fact, the postmortem microbiome richness differed notably across the analyzed body regions, as already proved by studies concerning microbial ante and postmortem investigations in humans (Figures 6, 7, 8) (Consortium HMP, 2012). Bacteroidetes and Actinobacteria were the least abundant phyla within the community and were excluded from the oral cavity and mainly detected on the skin and inferior side of the rabbit carcasses confirming the close association of these taxa to the environment. In contrast to Pechal and coworkers, we observed that Actinobacteria were more abundant on the side lying on the soil rather than on the exposed skin (Pechal et al., 2014). Actinobacteria slightly decreased over the decomposition suggesting that a kind of interaction and competition between exogenous and endogenous death‐related microbial communities occurred. The insects may have a strong effect on this aspect with colonizing flies importing exogenous bacteria (Weatherbee et al., 2017). The larvae play an important role as well, performing the extra‐oral digestions, conditioning the bacterial decomposing community.

Members of the phylum Bacteroidetes have colonized all types of environments on Earth and cultivated strains have been isolated from a broad and various selection of sources and soil samples including cultivated fields, greenhouse fields, and unexploited areas (Thomas, Hehemann, Rebuffet, Czjzek, & Michel, 2011). Environmental Bacteroidetes, including Flavobacteria, Cytophagia, and Sphingobacteria classes are thought to be specialized in the degradation of complex organic matter in the biosphere. Coherently with this observation and as documented by Roesch et al. (2007) about soil microbial diversity, our findings reveal the presence of Flavobacteriaceae and Sphingobacteriaceae families as the majority of Bacteroidetes.

## 5 CONCLUSIONS

This research describes the postmortem microbial communities developed on rabbit carcasses and discuss for the first time the effect of the fur on the colonization processes by microorganisms and insects. We demonstrated that the bacterial communities changed during the time with significant differences depending on the anatomical regions but independently of the presence of the fur.

These results are in agreement with previous studies and provide a better insight into the ecology of carrion decomposition, focusing on the novelty that neither entomofauna nor microbial communities are affected by the fur condition of the animals. Despite being preliminary, these observations allow to define some rules for further research, but as well for the applicability of the microbiome analysis in real cases:

- because of the absence of a fur effect, data obtained from human and pigs models, largely used in microbiome studies, can be applied in Forensic Veterinary investigations;
- size plays an important role in the rate of decomposition and insect colonization, but has little influence on the bacterial communities’ assemblage;
- water content is vital for the necrobiome development, therefore, analysis should take the content of water of the carcass/cadaver into account;
- necrobiome changes with time providing a useful tool for PMI estimation in support of Forensic Entomology analysis, however, a standardized protocol, which precisely defines the anatomical region of sampling(s), is required.

In addition, despite the results being promising so far, further experiments involving seasonal trials will have to be performed. Moreover, a standardized statistical model will be vital to create a bridge between academic research work and forensic investigators.

## CONFLICT OF INTEREST

None declared.

## AUTHORS CONTRIBUTION

Conceptualization: Stefano Vanin; formal analysis: Fabiola Tuccia, Stefano Vanin, Emad Zurgani; investigation: Emad Zurgani, Fabiola Tuccia, Sara Bortolini; project administration: Stefano Vanin; supervision: Stefano Vanin; writing—original draft publication: Fabiola Tuccia, Stefano Vanin; writing—review and editing: Fabiola Tuccia, Stefano Vanin.

## Data Availability

https://www.ncbi.nlm.nih.gov/sra/PRJNA521151

## APPENDIX 1

**Table:** TABLE A1 Initial weight of carcasses

Type	Carrion number	Weight (kg)
NF	1	3.1
	3	2.75
	5	2.85
F	2	3.45
	4	3.5
	6	3.45

**Table:** TABLE A2 18 processed swab samples for pyrosequencing analysis and the functional activity recorded (%)

Date	Decomposition stage	Day Exp	ADD	Body region	NF carrions functional activity (%)	F carrions functional activity (%)
25 March 2015	Active decay	8	50	Oral cavity	87.1	100.0
				Skin	45.2	25.8
				Soil/carrion	83.9	77.4
20 April 2015	Advanced decay	34	294	Oral cavity	22.6	71.0
				Skin	3.2	3.2
				Soil/carrion	54.8	58.1
18 May 2015	Dry	62	586	Oral cavity	3.2	87.1
				Skin	6.5	0.0
				Soil/carrion	12.9	64.5
